# Supplementary material for: The interactome of intact mitochondria by cross-linking mass spectrometry provides evidence for coexisting respiratory supercomplexes
Source: Mol Cell Proteomics. 2017 Dec 8;17(2):216–32. doi: 10.1074/mcp.RA117.000470 (PMC5795388; doi:10.1074/mcp.RA117.000470)
Supplement: Supplemental Data [file supp_RA117.000470_133922_0_supp_23978_fzffwf.zip › spectra_annotation/mito_DR_spectra_annotation/456-1-12-1-7-1.pdf]

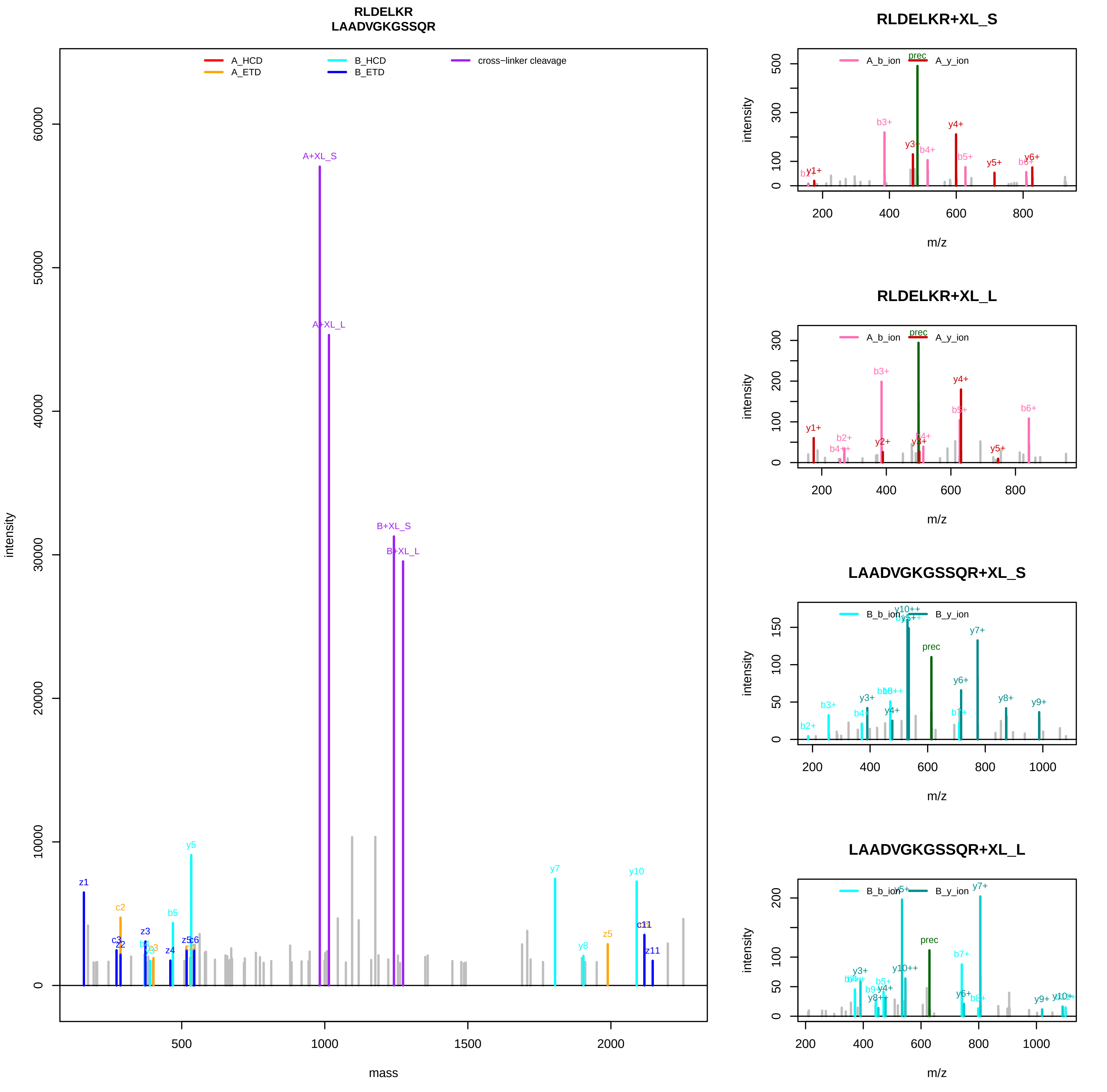
RLDELKR
LAADVGKGSSQR
A_HCD
A_ETD
B_HCD
B_ETD
cross−linker cleavage
A+XL_S
A+XL_L
B+XL_S
B+XL_L
z1
c2
z3
c3
z2
b5
y5
z4
y7
y8
z5
y10
c11
z11
intensity
mass
0
10000
20000
30000
40000
50000
60000
500
1000
1500
2000
RLDELKR+XL_S
RLDELKR+XL_L
LAADVGKGSSQR+XL_S
LAADVGKGSSQR+XL_L
A_b_ion
A_y_ion
B_b_ion
B_y_ion
prec
b3+
y3+
b4+
y4+
b5+
y5+
y6+
y1+
b6+
b2+
y2+
b4++
y10++
y7+
y6+
y8+
y9+
b7+
b3+
y3+
b5+
b8+
y10+
m/z
intensity
200
400
600
800
1000
